# Applying Newly Identified Financially Focused Home Care Quality and Performance Indicators In Alberta's $23.5B/annum Health System - Preliminary Findings

**DOI:** 10.23889/ijpds.v9i5.2493

**Published:** 2024-09-18

**Authors:** Max Jajszczok, Hude Quan, Catherine A Eastwood, Mingshan Lu, Ceara Cunningham

**Affiliations:** 1 University of Calgary

Through a multi-phased mixed-method research program including an international scoping review and a modified-Delphi panel with expert policy/academic/operational representatives from across Canada, three health-system, financially focused quality and performance home care indicators were identified.

This presentation will provide preliminary results of the application of the three financial indicators on overall health-system measures, system utilization and population outcomes within the province of Alberta. The time-period covered is the last five years data leading up to the start of the deceleration of the covid-19 global pandemic.

Based on provincial annual financial reporting, baseline 2016/17 fiscal year home care expenditures were $585K, this rose to significantly to $716M for fiscal year 2019/20, with the most significant increase (13%) occurred between the 2017/18 and 2018/19 years. Prior to 2016/17 home care expenditures increased at a rate less than 3%. These significant home care investments increased the percent of the overall expenditures in Alberta from 3.94 to 4.6%. Furthermore, the average annual expenditures per home care client rose from $5,417 to $6,112.

During this same time-period, numerous health-system indicators shifted to more favorable positions, with notable decreases in acute care days prior to death, number of those waiting in hospital for LTC, and preventing/delaying the need for LTC overall.

Based on comparisons of home care investments on the overall health-system and key health-system performance indicators focused on measuring system efficiencies, preliminary examinations suggest that significant home care focused investments in Alberta significantly improved health-system performance, efficiencies, and overall outcomes for the population.

